# Influence of sport mouthguards on the ecological factors of the children oral cavity

**DOI:** 10.1186/1472-6831-14-97

**Published:** 2014-08-05

**Authors:** Simonetta D’Ercole, Diego Martinelli, Domenico Tripodi

**Affiliations:** 1 Departments of Sperimental and Clinical Sciences, Dental School, University “G. D’Annunzio” of Chieti-Pescara, Chieti, Italy; 2 Departments of Medical, Oral, and Biotechnological Sciences, Dental School, University “G. D’Annunzio” of Chieti-Pescara, Chieti, Italy; 3 Unit of Pediatric Dentistry, Departments of Medical, Oral, and Biotechnological Sciences, Dental School, University “G. D’Annunzio” of Chieti-Pescara, Chieti, Italy; 4 Oral Microbiology, Department of Sperimental and Clinical Sciences, University “G. d’Annunzio” Chieti- Pescara, Via dei Vestini, 31 66100 Chieti, Italy

**Keywords:** Sport mouthguards, Dental devices, Salivary test, Streptococcus mutans

## Abstract

**Background:**

The use of fixed and/or removable dental devices is an attributable factor that may affect the oral cavity homeostasis. The aim of this study was to monitor the oral environmental changes caused by dental devices, as sports mouthguards with the aid of a chair-side test.

**Methods:**

Sixty children with sports-mouthguards were analyzed at baseline (T0), after 6 months of dental devices use (T1), after a year (T2) and after almost 6 months without using it (T3). At T0, a clinical monitoring was performed and the DMFT index was recorded. At each time of observation, the following parameters were recorded: FMPS, FMBS, unstimulated-flow rate, saliva consistency, resting pH, stimulated saliva, buffer capacity, the CFU/ml of *Streptococcus mutans*.

**Results:**

In 60 subjects, mean age 9.9 ± 1.2, mean value of DMFT 1.55 ± 1.29,dmf-t 3.43 ± 1.21, FMPS and FMBS values increased significantly at T2. The values of unstimulated flow rate vary significantly within the observation times. The pH value and the buffering capacity reduced significantly at T2. The tests for the detection of *S. mutans* were negative in all the subjects in several observation times. All patients regularly used fluoridated toothpaste and comply with normal standards of oral hygiene; but over time the patients lost their initial motivation.

**Conclusions:**

Sport treatment with dental devices dues to changes in oral ecological factors: increases FMPS, FMBS and reduces the buffering capacity and the salivary pH. The use of removable devices increases the retentive plaque surfaces and inhibits the protective effect of saliva.

The so-called “chair-side” tests were able to easily monitor patients and to determine the risk of oral disease during sport treatment.

## Background

The dental biofilm is a complex structure whose formation is influenced by many factors, which can alter both the structure and formation process. Normally the plaque coexists in the oral cavity in a sort of balance between activities of the microorganisms and host defenses. Pathogens can destroy this balance, which if disturbed can move towards the two main oral diseases: dental caries and periodontal disease. The use of fixed and/or removable dental devices is an attributable factor that may affect the balance. Sport mouthguards were dental devices that have been utilized by athletes who recognized the need for oral protection during their sports activities and Ethylene Vinyl Acetate (EVA) has become widely accepted as a mouthguard material [1-4].

Recent studies of hockey and football players have shown that “boil and bite” protective athletic mouthguards harbor a range of pathogenic and opportunistic bacteria, yeasts, and molds [5,6].

Ultrastructural analyses by SEM reveal that the microorganisms can be found both on the surfaces and in the porosities of the polymerized boil and bite mouthguard material [7]. A recent study over the course of a season confirmed that football mouthguards increased the number and severity of oral mucosal injuries [5,7]. Previous studies found that some commercially available mouthguards were contaminated before use [5].

No data are availables about the association between custom-made sport mouthguards and the changes occurring in the oral ecosystem; on the contrary several studies have been performed on patients wearing removable and fixed orthodontic appliances [8-10]. The relationship between the use of orthodontic appliances and the development of caries, however, is still under discussion. Some authors reported that the orthodontic treatment alone can not increase the incidence of caries [11,12] while others demonstrated an increase of enamel lesions or proximal caries in correlation with the use of fixed or removable devices [13-15]. Certainly, the orthodontic treatment involves the changes in oral microflora, increasing the cariogenic microorganisms in plaque and saliva. The effect of fixed orthodontic appliances on the colonization of cariogenic microorganisms has been well established; on the contrary it is not clear whether the removable appliances can increase the levels of pathogenic Streptococci in the oral cavity [16,17]. Few studies reported about the association between orthodontic treatment and the degree of salivary flow, and the buffering capacity of saliva, and still cover all fixed orthodontic treatment [8-10,18,19]. There is now ability to easily monitor patients and to determine the risk of caries and periodontal disease, before and during sport treatment, thanks to the introduction of the so-called “chair-side”tests. They allow identifying changes that occur in the oral cavity during treatment and are mainly based on research of *Streptococcus mutans* and *Lactobacillus* spp and determination of salivary characteristics (pH, buffer capacity, etc.) [20-24].

The purpose of this study was to monitor environmental changes of the oral cavity by the determination of clinical, salivary and bacterial markers, before, during and after sport treatment with mouthguards, with the aid of chair-side test.

## Methods

### Study population

60 children were selected, 27 males, 33 females, aged between 8 and 12 years, referred to the Department of Medical, Oral and Biotechnological Sciences, University of Chieti, in the period 2010–11.

The following inclusion criteria were considered:

1. Age: 7–14 years (7 < age <14)

2. Absence of any active carious lesion

3. Dental Care not in progress

4. Need for sport treatment with mouthguards.

Patients were excluded from the study if they met any of the following exclusion criteria:

1. Periodontitis

2. Partial or total removable prosthesis

3. Poor medical conditions (diabetes, asthma), systemic antibiotics, or local antimicrobials during 3 months preceding the sport treatment placement, patients under current medication affecting the saliva flow rate.

During the study, a patient who started a medication affecting the saliva flow rate or the biofilm composition was immediately excluded.

The selected subjects participated voluntarily in the study. Patients and their parents were first given oral and written information on the study’s purpose. Informed consent was given by signing a protocol. (Privacy Law DL 196/2003). In this study, the approval from the Ethical Committee it’s not reported because that’s not required for works that are based on research protocol on medical devices already used in the clinical protocols approved from the Department for the medical use.

A self-administered questionnaire was used to obtain data concerning a pathological complete history, a history of hard and soft tissues of the oral cavity, a family history, oral hygiene practices, fluoride intake and snacking habits (use of supplements, dietary information, such as intake of drinks, fruit juices and consumption of chocolates).

### Clinical monitoring

Patients were analyzed at baseline, before starting sport therapy with custom-made EVA mouthguards (T0), after 6 months of therapy (T1), after a year (T2) and finally after almost 6 months without using the device (T3).

At T0, a clinical monitoring was performed and on each patient were recorded the number of decayed (D), missing (M) and filled (F) teeth (T) (DMFT) and dmft to assess caries prevalence according to WHO criteria [25]. In addition, an oral examination of intraoral mucosal was performed and assessed the presence/absence of bad habits and/or parafunctional habits. During the first visit were explained to each patient the rules for a proper use of toothbrush and toothpaste. Each patient has repeated the indications received so the clinician was able to correct any inaccuracy.

At each time of observation, the following parameters were recorded: FMPS (Full Mouth Plaque Score) and FMBS (Full Mouth Bleeding Score) to evaluate the oral hygiene and periodontal status. The patients avoided eating or drinking and they didn’t effect the tooth brushing at least 2 hours before taking the samples in all stages. All procedures were done by one calibrated researcher, and commercial kits were used according to the manufacturer’s instructions.

### Saliva analysis

Then on each patient, in the 4 time points, the following indices were determined with the aid of the GC Saliva Check Kit (GC Corp., Belgium). The first was performed by a visual inspection of level of hydration. The unstimulated flow rate was measured visually, noting the time taken for a salivary droplet to form on the lower lip. A time greater than 60 s was considered as abnormally low (according to the manufacturer’s instructions). Then visually was analyzed the saliva consistency in the oral cavity. A sticky frothy saliva residue was considered as increased viscosity.

Subjects were then asked to pool their saliva in the floor of the mouth and then expectorate over 30 s into the collection cup. A pH tests trip was dipped in the saliva and the colour used to estimate the pH comparing with the testing chart available in the kit. To stimulate saliva it was given to the subjects a paraffin wax to chew and saliva was collected for 5 min in a measuring cup and the volume was calculated. From the collected saliva a drop was placed on a buffering strip, left for 5 min and then measured on the pH scale provided.

### Bacterial markers

To estimate the number of colony-forming units of *Streptococcus mutans* counts (SM) per milliliter of saliva (CFU/mL) GC Saliva-Check Mutans (GC Corp., Belgium)was used.

This test kit provides a semi-quantitative evaluation of the level of *S.mutans* in the saliva in 15 minutes by using monoclonal antibodies.

### Statistical analysis

For statistical analysis the Matched-Pairs Signed-Wilkoxon-rank test have been used. Data are shown as Mean values (Standard Deviation-SD), and for all analyses a P-value of less than 0.05 was considered significant.

## Results

**Figure 1:**
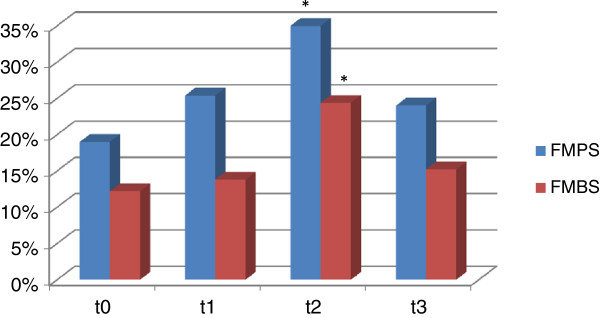
Periodontal indices of studied population. *p < 0.05 compared to T0.

As shown in Table 1, sixty subjects, mean age 9.9(1.2), with mean value of DMFT 1.55 (1.29) and dmf-t 3.43 (1.21) participated to this study. Allergies, in particular to nickel and resins-define, which could affect the individual response, were almost absent. In addition, information obtained indicated that none of the 60 children was suffering from food allergies or medication and only one of them had undergone surgery. All patients were not receiving regular fluoride but they regularly used fluoridated toothpaste. From the data obtained about the eating habits, it was shown that the majority of subjects took regular carbohydrates in the diet (pasta, bread, cakes, candies, etc.), but it doesn’t abuse of it (data not shown).With regard to clinical indices (Figure 1), the FMPS value increased significantly from T0 to T2 and then restabilized at T3, about to the levels of T1. The value of FMBS unchanged between T0 and T1 and reached values statistically significant at T2 and newly improved at T3.

**Table 1:** Demographic characteristics of the studied population

	**T0**
**Average age**	**9.9 (1.2)**
**Sex**	**27 M 33 F**
**DMFT**	**1.55 (1.29)**
**dmft**	**3.43 (1.21)**
**Allergies**	**Yes 20%**
**Sealing**	**Yes 40%**
**Intake of fluoride**	**No**
**Use of fluoridated toothpaste**	**Yes 100%**

**T0 =** baseline.

**Figure 2:**
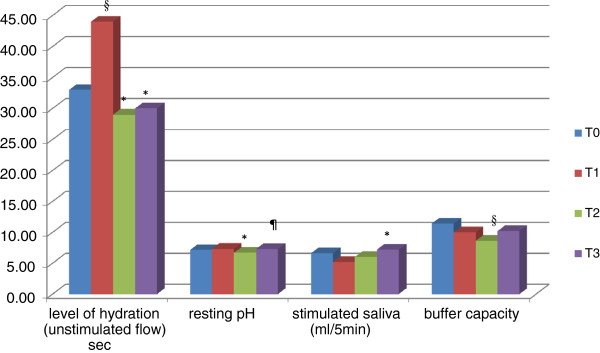
**Salivary and microbiological factors examined with the aid of a chair-side salivary test. T0** = baseline. **T1** = patients carrying the sport mouthguards from 6 months. **T2** = patients carrying the sport mouthguards from1 year. **T3** = patients who completed sport therapy for 6 months. ^§^**p** < 0.05 compared to T0. *p < 0.05 compared to T1. ^¶^**p** < 0.05 compared to T2.

The results of saliva and bacteria analysis are shown in Figure 2 and Table 2. Within the four observation times there was a significant variation of the values of unstimulated flow rate, who suffered a statistically significant increase to T1, and a statistically significant reduction at T2 and T3 compared to T1, so as to return to baseline values. The saliva consistency does not undergo significant changes over time. The pH value of the baseline, however, undergoes a significant decrease in T2 to return to the previous levels in T3. The amount of saliva produced under stimulation in 5 minutes didn’t vary significantly at T0, T1 and T2, but the difference between T1 and T3 was statistically significant. The buffering capacity was drastically reduced in a statistically significant way at T2.

**Table 2:** **Salivary consistence and presence/absence of *S. mutans *** **in the population studied**

	**T0**	**T1**	**T2**	**T3**
Saliva consistency	Normal	Normal	Increased in 20% of cases	Increased in 20% of cases
*Streptococcus mutans*	Negative	Negative	Negative	Negative

**T0** = baseline.

**T1** = patients carrying the sport mouthguards from 6 months.

**T2** = patients carrying the sport mouthguards from1 year.

**T3** = patients who completed sport therapy for 6 months.

The tests for the detection of *Streptococcus mutans* were negative in all the subjects on several observation times (Table 2).

The Table 3 shows that patients comply with normal standards of oral hygiene, both in regard to brushing the teeth that cleaning removable appliance that actually improved during the first months of therapy, but over time the patients lost their initial motivation (see difference between T1 and T2).

**Table 3:** Oral hygiene habits of the studied population

	**T0**	**T1**	**T2**	**T3**
Brushing/die	2 times.	2.2 times.	1.6 times.	1.8 times.
Cleaning device/die	ND	2.2 times.	1.6 times.	ND

**T0** = baseline.

**T1** = patients carrying the sport mouthguards from 6 months.

**T2** = patients carrying the sport mouthguards from1 year.

**T3** = patients who completed sport therapy for 6 months.

**ND**: Not Detected.

## Discussion

This longitudinal study shows the clinical, salivary and bacterial changes that occurred in the oral cavity during and after treatment with custom-made sport mouthguards. Normally, the oral cavity has the ability to adapt it to the presence of a foreign body, increasing the salivary flow which contributes to autoclysis and altering the composition of saliva to maintain high values of pH and buffering capacity, so as to prevent the colonization of microorganisms potentially pathogenic by the refusal of the optimal environmental conditions.

The advent of a simple chair-side saliva check kit allows to easily monitor patients and to determine the risk of caries and periodontal disease before and during sport treatment.

The unstimulated salivary flow, as observed in this study, increased initially after 6 months of therapy in response to a foreign body, but after 1 year of therapy decreased significantly. The amount of saliva produced under stimulation in 5 minutes (salivary flow) didn’t vary significantly between the baseline and during all the treatment. In contrast, 6 months after the end of treatment there was a statistically significant increase compared with 6 months of therapy, confirming the results obtained from Sanpei and colleagues [19] who showed how changes in salivary flow that occur in the period of orthodontic treatment were statistically insignificant. On the contrary Chang and colleagues [8], Ulukapi and colleagues [18], Cheng and colleagues [9] showed that the salivary flow increased significantly after the orthodontic devices positioning.

The results of this study demonstrated that the use of removable appliances, as custom-made mouthguards, lead to a significant lowering of the pH scale defined in moderately acid after a year of therapy, necessitating later than 6 months after therapy to return to normal levels. The pH decreased as the mouthguard increased the areas and retentive surfaces against the plaque, thus causing high acid levels due to high concentration of hydrogen ions in the oral cavity.

The buffering capacity of saliva prevents the colonization of pathogenic microorganisms in the mouth and neutralizes acids produced by acidogenic bacteria, thus preventing the demineralization of enamel. This can be regarded as one of the best indicators of risk, as revealed bythe answer of the host [10]. Patients with high buffering capacity are often resistant to the development of caries, because the high response of the host can be compensatory against the activity of the lesion.

Studies on the salivary markers and their association with the prevalence of caries in adult populations have been widely performed in the past, but with mixed results [8,9,18,19,26].

However, there seems to be a correlation between the saliva pH and the prevalence of early carious lesions, as well as between the buffering capacity and potential activity in lesions of moderate type. Varma and colleagues [26] reported that the pH of the saliva can be considered a predictive factor in the development of pre-carious lesions. In the presence of active lesions, however, the buffer capacity can be of help in predicting the potential activity in the dentinal lesions present.

In this study, the buffering capacity of saliva was greatly reduced in a statistically significant way after a year of sport treatment with mouthguards.

The results showed that the dental devices does not influence the salivary flow, which has among other things, the function of providing the bicarbonate ions and thus to increase the pH. On the contrary, in this case, in parallel, also the pH decreases as well as the buffering capacity, showing an altered response of the host in maintaining oral health in adverse situations and thus decreasing the protective and preventive activity of the saliva.

About the effect played by removable appliances, Batoni and colleagues [27] showed that the presence in the oral cavity of orthodontic removable devices leads to the creation of new retentive areas and surfaces adhesion- and growth-promoting of *S. mutans*.

Glass and colleagues reported that the boil and bite mouthguards were contaminated by microorganisms and have the propensity to become a microbial reservoir. Therefore, they have the potential to produce oral and systemic diseases because the jagged, sharp areas of the posterior regions of typical mouthguards are in close proximity to the pterygoid plexus of veins and, so, the entire circulatory system [5-7].

In this study the use of the test “Saliva-Check Mutans GC” showed that levels of *S. mutans* remain unchanged during the study, in agreement with Sanpei and colleagues [19] and Lara-Carrillo and colleagues [10]. The test used in this study remained negative for all time, but the test in question presented low sensitivity and low quantitative discrimination, because based only on the presence/absence of the microorganism with level of cut-off equal to 5 × 10^5^ CFU/ml.

The microbiological test used in this study was easy to use and relatively inexpensive and thus it allows realizing a quick screening of populations at risk due to its own cut-off of reference, even if its accuracy was lower than that of the conventional cultivation method [28-30].

The use of sport mouthguards lead to a worsening of the oral health of analyzed patients, as demonstrated by a significant increase after one year of therapy of the clinical indices FMPS and FMBS, which would significantly improve with the suspension for 6 months of the sport treatment. The increase of the bacterial plaque was due both to changes in the characteristics of the saliva, which we have seen was able to facilitate the formation of the biofilm, both to the particular features of the device. The plaque has a tendency to accumulate in the retentive areas of springs, claps and polymeric-material baseplate [7,31] that are sites where it can easily be the penetration of microorganisms due to the properties of the material itself. The EVA is a copolymer and when the material is prepared it’s possible to observe the reactions monomer-polymer. If the process is not performed correctly, pores can appear on the surface facilitating the adhesion of microorganisms [1-3,32].

Based on these results, several preventive strategies for the changes that occur in the oral cavity of athletes should be implemented. The study of the quality, pH and buffering capacity of the saliva, the monitoring of the bacterial count, plaque and gingival inflammation can be an important part of a complete clinical control. Maintain a good oral hygiene during sport treatment is very difficult. Anyway in light of the possible consequences (development of tooth decay, gingival and periodontal disease) is crucial to give the patient proper oral hygiene instructions starting therapy and to monitor the course of therapy. Then, the tests must be repeated at regular intervals during treatment and the patient must be motivated with regular reminders of oral hygiene and controls in order to avoid the onset of disease, since, as demonstrated by this study, the motivation is lost in time and it’s reduced the number of washes of teeth and device. Other preventive approaches require that the patient is informed about the potential of cariogenic foods and beverages and use of appropriate antimicrobial therapy and administration of fluoride. According to Benson and colleagues [33] the subministration of fluoride 0.05% through mouthwashes and topical application in the gel can help to prevent the development of the biofilm, but as demonstrated in this study in which patients were regularly using fluoride toothpaste, there is no influence on the ecological characteristics of the oral cavity. It should be recommended in any case the use of chlorhexidine, due to its widely demonstrated bactericidal activity. Disinfection with antimicrobial agents should obviously include not the onlyoral cavity but also the device [34,35]. Alternatively, as suggested by Arnold and colleagues [36] chlorhexidine can be incorporated in the matrix EVA that slowly released the antimicrobial agent into the oral cavity.

## Conclusions

Sport treatment with custom-made mouthguards due to changes in ecological factors of the oral cavity, because increases FMPS and FMBS and reduces the buffering capacity and salivary pH, thus inhibiting the protective effect of saliva. Furthermore, after a year of therapy there is a worsening of the clinical indices of the patient, who loses the motivation to maintain good oral hygiene. It’s necessary to identify those at risk from the outset of therapy, and then still to monitor patients during all therapy.

The advent of simple chair-side check kits that allow to analyze the bacterial count, the labial hydration level of unstimulated saliva, quality, resting pH and buffering capacity of stimulated saliva, to be associated with clinical indices, necessary for the determination of plaque and gingival inflammation, it offers the possibility to follow very well during the time patients with sport mouthguards without incurring in future diseases.

## Abbreviations

EVA: Ethylene Vinyl Acetate; DMFT: Number of decayed (D), missing (M) and filled (F) teeth (T); FMPS: Full mouth plaque score; FMBS: Full mouth bleeding score; CFU/mL: Number of colony-forming units per milliliter; SD: Standard deviation.

## Competing interests

The authors declare that they have no competing interests.

## Authors’ contributions

SD has written the clinical protocol and the manuscript. DM has done the salivary test on the patients. DT has organized the research. All authors read and approved the final manuscript.

## Authors’ information

SD, DDS, PhD, is a research fellow. DM, DDS, is a postgraduate student. DT, MD, DDS is a professor.

## Pre-publication history

The pre-publication history for this paper can be accessed here:

http://www.biomedcentral.com/1472-6831/14/97/prepub
